# Une maladie d’Ebstein asymptomatique découverte à l’occasion d’une douleur thoracique atypique: à propos d’un cas

**DOI:** 10.11604/pamj.2022.43.118.33505

**Published:** 2022-11-01

**Authors:** Khaoula Bourzeg, Moulay Achraf Choukri, Rim Zerhoudi, Assala Cherki, Abdelmajid Bouzerda, Ali Khatouri

**Affiliations:** 1 Service de Cardiologie, Hôpital Militaire Avicenne de Marrakech, Université Cadi Ayyad, Faculté de Médecine et de Pharmacie, Marrakech, Maroc

**Keywords:** Maladie d’Ebstein, valve tricuspide, cardiopathie congénitale, cas clinique, Ebstein´s anomaly, tricuspid valve, congenital heart disease, case report

## Abstract

La maladie d´Ebstein est une malformation congénitale rare dont la présentation clinique diffère selon la forme anatomique et l´âge du patient. Chez l´adulte, elle se présente essentiellement sous forme d´insuffisance cardiaque droite ou globale ou des troubles de rythme. La survie est exceptionnellement longue dans certains cas. La prise en charge varie selon la forme anatomique et la présentation clinique allant de la simple surveillance pour les patients asymptomatiques, à la prise en charge chirurgicale pour les autres cas. Nous rapportons le cas d´un patient ayant une maladie d´Ebstein asymptomatique jusqu´à l´âge de 54 ans où elle est découverte à l´occasion de douleurs thoraciques atypiques, présentation non commune de la maladie. La prise en charge dans ce cas a consisté dans ce cas en une surveillance clinique et échographique stricte.

## Abstract (en)

Ebstein´s disease is a rare congenital malformation whose clinical presentation differs according to the anatomical form and age of the patient. In adults, it presents mainly as right or global heart failure or arrhythmia. Survival is exceptionally long in some cases. Management varies according to the anatomical form and clinical presentation, ranging from simple surveillance for asymptomatic patients to surgical management for other cases. We report the case of a patient with asymptomatic Ebstein´s disease until the age of 54 years when it was discovered at the time of atypical chest pain, an uncommon presentation of the disease. Management in this case consisted of strict clinical and ultrasound surveillance.

## Introduction

L´anomalie d´Ebstein est une malformation congénitale rare de la valve tricuspide qui intéresse 1>/20 000 naissances vivantes et représente moins de 1% de tous les cas de cardiopathie congénitale [1]. Elle a été décrite pour la première fois par Wilhelm Ebstein en 1866 [2]. Elle est présente à la naissance mais les symptômes peuvent survenir à tout âge, avec une espérance de vie moyenne de la troisième décennie, comme indiqué dans un rapport précoce [3]. La présentation clinique varie selon la période de découverte, allant de la forme néonatale, très grave, aux formes mieux tolérées de l´adolescent et de l´adulte [4]. A l´âge adulte, elle se manifeste typiquement par une arythmie ou une insuffisance cardiaque droite ou gauche [5]. Nous rapportons le cas de notre patient vue l´évolution silencieuse de la maladie d´Ebstein chez lui jusqu´à l´âge de 54 ans et surtout vue la présentation clinique atypique en dehors de l´insuffisance cardiaque et l´arythmie.

## Patient et observation

**Informations du patient:** il s´agit d´un patient de 54 ans, ayant comme facteur de risque cardiovasculaire une obésité abdominale, âge et le sexe masculin et comme antécédent toxico-allergique un tabagisme chronique à 10 PA sevré il y a 30 ans, avait été admis au service de cardiologie de l´Hôpital Militaire Avicenne de Marrakech pour douleur thoracique atypique latéralisée du côté gauche irradiant vers le dos non rythmé par l´effort coté 3/10 à l´échelle visuelle analogique qui évolue depuis 4 jours avant son admission. Il n´avait pas de dyspnée, ni orthopnée, ni palpitations, ni autres signes fonctionnels associés. L´évolution a été marquée par la sédation de la douleur spontanément.

**Figure 1:**
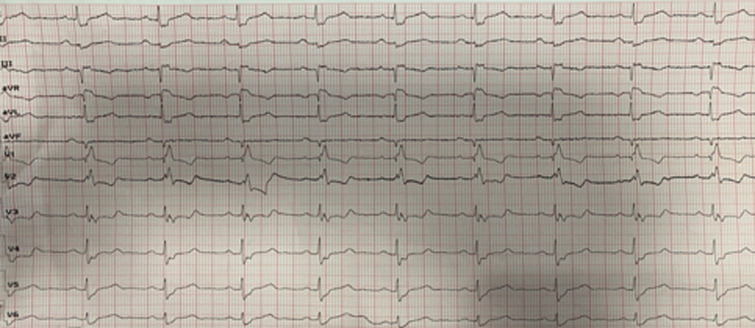
ECG objectivant des QRS larges avec un bloc de branche droit complet avec des troubles de repolarisation type sous décalage du segment ST avec des ondes T négatives en antéro-septal

Devant les facteurs de risque cardiovasculaires du patient une origine coronarienne est à éliminer immédiatement, donc un Electrocardiogramme (ECG) a été réalisé objectivant des QRS larges avec un bloc de branche droit complet et des troubles de repolarisation type sous décalage du segment ST avec des ondes T négatives en antéro-septal sans autres anomalies notables (Figure 1). Un complément par des troponines ultrasensibles s´est avéré nécessaire revenant négative à 2 reprises.

**Résultats cliniques:** sur le plan clinique, l´examen objective un patient conscient avec une tension artérielle à 120/80 mmHg symétrique aux 2 membres supérieures, une fréquence cardiaque à 62 bat/min, FR à 18 c/min, SaO_2_= 96% à l´air libre, dextro = 0,92 et des conjonctives normo-colorées, avec à l´examen cardiovasculaire des bruits de cœurs bien perçus et régulier associées à un souffle d´insuffisance tricuspide sans signes d´insuffisance cardiaque. L´examen pleuro-pulmonaire ainsi que le reste de l´examen somatique est sans particularités.

**Figure 2:**
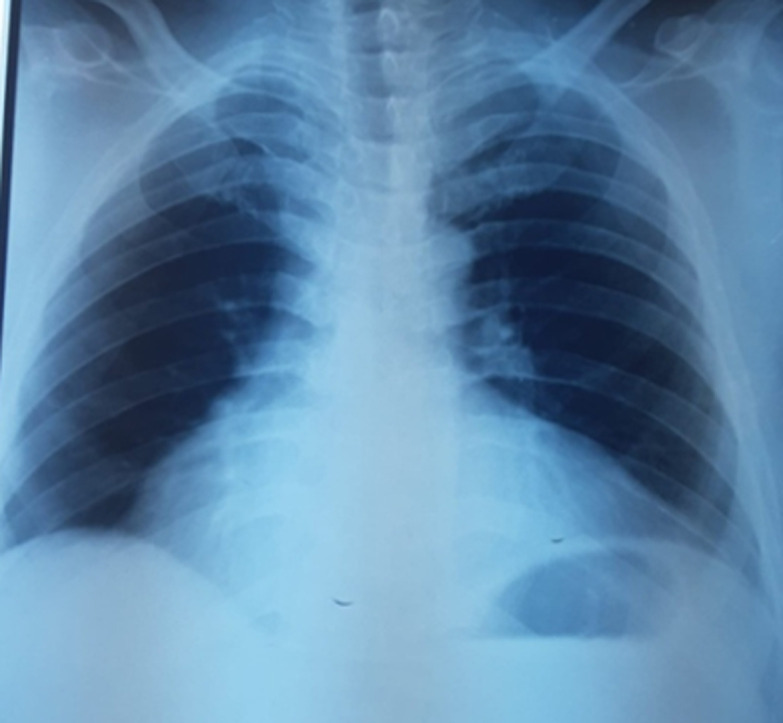
radio de thorax objectivant une cardiomégalie à pointe sous-diaphragmatique et index cardio-thoracique à 0,6 avec hyperconvexité de l’arc inferieur droit

**Démarche diagnostique:** à la radiographie thoracique de face, on notait une cardiomégalie à pointe sous-diaphragmatique et index cardio-thoracique à 0,6 avec hyperconvexité de l´arc inferieur droit. La cage thoracique et le parenchyme pulmonaire étant sans anomalie (Figure 2).

**Figure 3:**
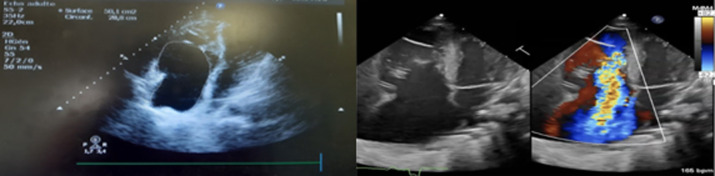
ETT objectivant une insertion basse du feuillet septal et postérieur avec dilatation importante de l’oreillette droite par atrialisation de ventricule droit

Le patient a bénéficié d´un complément par électrocardiographie (ETT) (Figure 3) objectivant une insertion basse du feuillet septal et postérieur de la valve tricuspide à 20mm en dessous de l´anneau tricuspide, le feuillet antérieur étant de mobilité réduite. Une dilatation importante de l´oreillette droite par atrialisation de ventricule droit (VD). Le VD rudimentaire et son moignon atrialisé sont dilatés et responsable d´une compression du VG. Insuffisance tricuspide massive et absence de barrage tricuspide. Sans autres malformations cardiaques associées. Donc au total, c´est une maladie d´Ebstein type C par attachement du feuillet septal et postérieur.

Sur le plan biologique, la fonction rénale et hépatique étaient normales, ainsi que tout l´ionogramme sanguin, de même que la protidémie et l´albuminémie. Le bilan des facteurs de risques incluant un bilan lipidique et une HB1Ac et acide urique était sans anomalies. Dans notre cas, avant de confier la douleur thoracique à un angor fonctionnel, un complément par épreuve d´effort a été fait revenant négatif cliniquement et électriquement. Un holter ECG à la recherche d´éventuelle arythmie a été fait revenant sans anomalies.

**Intervention thérapeutique et suivi:** le traitement dans ce cas était conservateur avec une surveillance rapprochée clinique et échocardiographique. L´évolution était marqué par la récurrence de douleur thoracique mais qui restent tolérée, non gênante ni limitante de l´activité quotidienne et le patient refuse tout acte chirurgical.

**Consentement éclairé:** un consentement éclairé écrit, daté et signé a été obtenu du patient ayant permis la réalisation de ladite exploration.

## Discussion

La maladie d´Ebstein est une anomalie congénitale de la valve tricuspide dont les feuillets septal et postérieur sont accolés à la paroi ventriculaire et déplacés vers la pointe du ventricule droit [1,6]. Ces anomalies anatomiques divisent le ventricule droit en une partie proximale à paroi mince qui s´atrialise et s´élargit, et un composant trabéculé plus distal qui forme le ventricule droit fonctionnel [7]. Les anomalies retrouvées peuvent être expliquées embryologiquement. En fait, le feuillet antérieur se développe en premier à partir du mésenchyme entourant l´orifice atrio-ventriculaire, puis les feuillets postérieur et septal se développent par délamination du myocarde. Dans le cas de l´anomalie d´Ebstein, il y a un défaut de délamination, laissant ainsi l´ensemble des feuillets septale et postérieure ainsi que la partie distale du feuillet antérieur en bas à l´intérieur ou adhérents au ventricule droit [6].

Les conséquences physiopathologiques de cette malformation sont une insuffisance tricuspide, une désynchronisation mécanique intra-atriale par activation séquentielle du segment atrial vrai et du segment ventriculaire atrialisé et donc une diminution du flux antérograde et de la pré-charge du ventricule gauche, ce qui peut expliquer l´angor rapporté par notre patient. Les malformations cardiaques souvent associées à l´anomalie d´Ebstein sont la communication interauriculaire dans 80% à 94% des cas (CIA) [8], le foramen ovale persistant, la sténose ou une atrésie pulmonaire, et la Communication interventriculaire (CIV) [9]. Chez 39% des patients, une atteinte myocardique ou valvulaire gauche était associés à l´anomalie de base [9]. Ainsi, la variation anatomique de la valve tricuspide dans l´anomalie d´Ebstein augmente le risque de connexions auriculo-ventriculaires accessoires et de pré-excitation, et les études rapporte que 6 à 36% des patients ont des voies accessoires [10].

La présentation clinique de l´anomalie d´Ebstein est variable, mais elle est diagnostiquée généralement à l´occasion d´une cyanose, une insuffisance cardiaque droite ou une arythmie. Tandis que la cyanose et l´insuffisance cardiaque droite sont plus fréquentes dans la population pédiatrique, l´arythmie est plus fréquente chez les adultes. Contrairement à notre cas où la découverte était guidée par des douleurs thoraciques. Et pour le diagnostic, l´échocardiographie bidimensionnelle reste un bon test initial.

**Figure 4:**
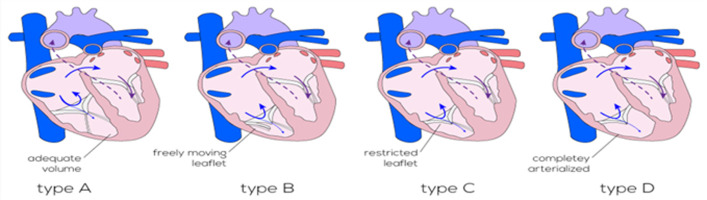
classification de Carpentier de la maladie d’Ebstein

Afin d´avoir une idée sur la sévérité de la maladie des classifications ont été établis. La première développée par Carpentier incluant quatre types: Type A (moins grave): les feuillets postérieure et septale sont déplacées apicalement, dysplasiques, ou absents. Le volume du VD est adéquat. Type B: les valves antérieure, postérieure, et septales sont présents, mais sont relativement petits et déplacés dans un mode de spirale vers l´apex. La chambre ventriculaire atrialisée est modérément grande. Type C (le cas de notre patient): la valve antérieure a un mouvement limité avec des cordages courts et fusionnés. Les valves postérieure et septale sont déplacées, dysplasique, et généralement pas reconstructible. La chambre ventriculaire atrialisée est grande. Type D (la plus grave): la valve antérieure est fortement déformée et déplacée dans la voie d´éjection du ventricule droit [1,11] (Figure 4). Une autre développée par Yuan [9] incluant quatre grades calculés à partir du rapport entre la surface combinée de l´oreillette droite et de la partie atrialisée du VD avec la partie fonctionnelle du VD et du cœur gauche a la coupe quatre caves à la fin de la diastole: grade 1: ratio <0,5; grade 2: ratio 0,5-0,99; grade 3: ratio 1-1,49 et grade 4: ratio >1.5.23. La gravité et la mortalité augmente avec le niveau de grade.

La prise en charge de la maladie d´Ebstein varie selon la forme anatomique et la présentation clinique. Les sujets ayant un déplacement mineur de la valve tricuspide sont souvent asymptomatiques et ne nécessitent pas de traitement particulier. Chez ces patients, une surveillance clinique et échographique régulière est requise. Elle recherchera une arythmie, une dilatation des cavités droites ou une dysfonction systolique ventriculaire droite [12] et c´est l´attitude adoptée chez notre patient. Pour les patients symptomatiques le traitement comprend deux volets: un volet médical et un volet chirurgical. Le traitement de l´insuffisance cardiaque droite implique un ajustement de la fréquence cardiaque et de la précharge et la restriction de l´exercice. Cela peut inclure un régime pauvre en sodium, des diurétiques oraux, de la digoxine et un inhibiteur de l´enzyme de conversion de l´angiotensine (ECA) à faible dose. La prise en charge de la poussée d´insuffisance cardiaque droite se base principalement sur les diurétiques, les vasodilatateurs, les cardiotoniques, le régime pauvre en sodium [9]. Une intervention chirurgicale est indiquée lorsque le patient devient symptomatique ou lorsqu´ils surviennent une arythmie ou des modifications échocardiographiques [13]. Elle consiste en une réparation ou en un remplacement de la valve tricuspide, associée ou non à une anastomose cavo-pulmonaire totale ou partielle [13].

## Conclusion

D´après notre cas, la maladie d´Ebstein peut se présenter sous forme d´un tableau atypique déroutant. Bien que les résultats échocardiographiques suffisent à diagnostiquer l´anomalie d´Ebstein, le rôle de l´imagerie cardiaque par rétrécissement mitral (RM) cardiaque est indispensable car elle fournit à la fois anatomie morphologique et fonctionnelle, ce qui nous aide à mieux comprendre les changements hémodynamiques et à la planification d´éventuelles techniques chirurgicales.
